# Simultaneous gene transfer of bone morphogenetic protein (BMP) -2 and BMP-7 by *in vivo *electroporation induces rapid bone formation and BMP-4 expression

**DOI:** 10.1186/1471-2474-7-62

**Published:** 2006-08-03

**Authors:** Mariko Kawai, Kazuhisa Bessho, Hiroki Maruyama, Jun-ichi Miyazaki, Toshio Yamamoto

**Affiliations:** 1 Department of Oral Morphology, Graduate School of Medicine, Dentistry and Pharmaceutical Sciences, Okayama University, Okayama 700-8525, Japan; 2 Department of Oral and Maxillofacial Surgery, Graduate School of Medicine, Kyoto University, Kyoto 606-8507, Japan; 3 Division of Nephrology and Rheumatology, Niigata University Graduate School of Medical and Dental Sciences, Niigata 951-8120, Japan; 4 Division of Stem Cell Regulation Research, Osaka University Medical School, Osaka 565-0871, Japan

## Abstract

**Background:**

Transcutaneous *in vivo *electroporation is expected to be an effective gene-transfer method for promoting bone regeneration using the BMP-2 plasmid vector. To promote enhanced osteoinduction using this method, we simultaneously transferred cDNAs for BMP-2 and BMP-7, as inserts in the non-viral vector pCAGGS.

**Methods:**

First, an *in vitro *study was carried out to confirm the expression of BMP-2 and BMP-7 following the double-gene transfer. Next, the individual BMP-2 and BMP-7 plasmids or both together were injected into rat calf muscles, and transcutaneous electroporation was applied 8 times at 100 V, 50 msec.

**Results:**

In the culture system, the simultaneous transfer of the BMP-2 and BMP-7 genes led to a much higher ALP activity in C2C12 cells than did the transfer of either gene alone. *In vivo*, ten days after the treatment, soft X-ray analysis showed that muscles that received both pCAGGS-BMP-2 and pCAGGS-BMP-7 had better-defined opacities than those receiving a single gene. Histological examination showed advanced ossification in calf muscles that received the double-gene transfer. BMP-4 mRNA was also expressed, and RT-PCR showed that its level increased for 3 days in a time-dependent manner in the double-gene transfer group. Immunohistochemistry confirmed that BMP-4-expressing cells resided in the matrix between muscle fibers.

**Conclusion:**

The simultaneous transfer of BMP-2 and BMP-7 genes using *in vivo *electroporation induces more rapid bone formation than the transfer of either gene alone, and the increased expression of endogenous BMP-4 suggests that the rapid ossification is related to the induction of BMP-4.

## Background

Non-viral gene delivery systems are potentially useful in gene therapies for tissue regeneration or repair [1,2]. In particular, electroporation is attractive, because it is an easy and inexpensive method that requires only a plasmid and a device for performing electroporation [3,4]. In addition, the method does not require viral vectors, expensive proteins, or carrier matrices. Previously, we constructed a human BMP-2 gene expression vector (pCAGGS-BMP-2) and showed that transferring the BMP-2 gene into rat skeletal muscles by *in vivo *transcutaneous electroporation induced ectopic bone formation [5]. However, there was no significant relationship between the dose of pCAGGS-BMP-2 plasmid vector used and the volume, quality, or time course of the ectopic bone formation. In this model, the surface area the electrodes can cover restricts the volume of the injected plasmid to 50 μl. Furthermore, any interaction between the plasmid dose and the electrical parameters can affect the efficiency of the gene transfer [6]. For clinical applications, it is important to optimize the method to enhance bone formation at the level of the intrinsic osteoinductive activity.

Comparative analyses of the osteogenic activity of various human BMP adenoviral vectors have indicated that each BMP has a different potential to induce bone formation [7,8]. In culture, protein purified from the supernatant of adenoviral vector-infected epithelial cells expressing both BMP-2 and BMP-7 accelerates the differentiation of preosteoblastic or premyogenic cells into osteoblastic cells [9]. In addition, culture supernatant from CHO cells that were transiently transfected with equal amounts of BMP-2 and BMP-7 expression vectors induces maximal alkaline phosphatase (ALP) activity in a mouse stromal cell culture system [10]. These researchers concluded that the combined transfer of the BMP-2 and BMP-7 genes to epithelial cells such as CHO or 293 cells produces the heterodimer BMP-2/7, and that it is the BMP-2/7 in the supernatant that enhances the differentiation of the preosteoblastic or myoblastic cells into osteogenic cells, leading to osteoinduction [9,10]. However, there are few reports of the effect on bone formation of the simultaneous and direct gene transfer of two or more BMPs. The present study was undertaken to determine whether the combined gene transfer of BMP-2 and BMP-7 into skeletal muscles in rats using *in vivo *electroporation could induce ectopic bone formation more rapidly than the transfer of only one of these genes. In addition, the endogenous BMP-4 mRNA expression levels and BMP-4-expressing cells were examined, since several reports show that exogenous BMPs elevate the levels of other BMPs or BMP-4 mRNA [11-14]. Prior to the *in vivo *study mentioned above, the effect of the combined direct gene transfer of BMP-2 and BMP-7 into myoblastic cells was also assessed using an *in vitro *gene-transfer system.

## Methods

### Plasmid vector

Human BMP-7 cDNA was obtained by PCR with pUC BMP-7 as the template and the following primers: human BMP-7 forward primer, 5'-GAG AGA GAG *AAGCTT *GGA TCC ATG GTG GCC GGG ACC CGC (ATG, initial codon); human BMP-7 backward primer, 5'-AGA GAG AG *AAGCTT *CTA GTG GCA GCC ACA GGC CCG GAC CA (CTA, stop codon). Both primers had *SwaI *recognition sites (italicized). The PCR protocol consisted of 25 cycles of 15 sec at 98°C, 2 sec at 65°C, and 30 sec at 74°C, with KOD DNA polymerase (ToYoBo, Osaka, Japan). The PCR product was blunt-ended and ligated into the *Eco*RI-digested and blunt-ended cloning site of the pCAGGS expression vector, which contains the CAG (cytomegalovirus immediate-early enhancer/chicken β-actin hybrid) promoter [15], to yield pCAGGS-BMP-7. The 1296-bp insert sequence was confirmed by DNA sequencing. PCAGGS-BMP-2 was described previously [5]. Plasmids were grown in *Escherichia coli *DH5α. Plasmid vectors were prepared using a Qiagen EndoFree plasmid Giga kit (Qiagen GmbH, Hilden, Germany), as described previously [4].

### ALP activity in C2C12 cells

We investigated the activity of ALP, which is a marker for osteoblastic differentiation, in C2C12 cells, a mouse myoblastic cell line, by directly and simultaneously transfecting them with equal doses of pCAGGS-BMP-2 and pCAGGS-BMP-7. The cells were cultured at 1 × 10^5 ^cells per well in 12-well plates in 1 ml growth medium (DMEM, 15% fetal bovine serum) for 1 day before the transfection. The next day, 0.5 μg pCAGGS and 0.5 μg pCAGGS-BMP-2, 0.5 μg pCAGGS and 0.5 μg pCAGGS-BMP-7, or 0.5 μg pCAGGS-BMP-2 and 0.5 μg pCAGGS-BMP-7 were diluted in 100 μl Opti-MEM (Invitrogen, California, USA), then mixed with 4.0 μl Lipofectamine 2000 (Invitrogen, California, USA) in 100 μl Opti-MEM. These DNA-lipofectamine 2000 complexes were added to each well of 90% confluent C2C12 cells. One day after the transfection, the medium was changed. At 10 days after the transfection, the cells were rinsed with phosphate buffered saline (PBS), and the cells from two 12-well plates were collected. The cells were lysed with 1 M Tris-HCl (pH 7.4), 5 M NaCl, 0.5 M EDTA, 0.5 M NaF, and 10% Nonident P-40. The lysates were spun and the supernatant collected. The quantitative ALP activity data and total protein content of the supernatants were determined by the p-nitrophenyl phosphate method. One 12-well plate was used for the histochemical staining of ALP, performed using a Sigma diagnostic ALP kit (Sigma, St. Louis, MO).

### Animals

Nine-week-old male Wistar rats were purchased from Kurea (Osaka, Japan) and maintained under specific pathogen-free conditions in our animal facility. All procedures were approved by the Animal Research Control Committee of Okayama University (approval No.oku-2005110).

### Intramuscular DNA injection and transcutaneous electroporation

**Figure 1:**
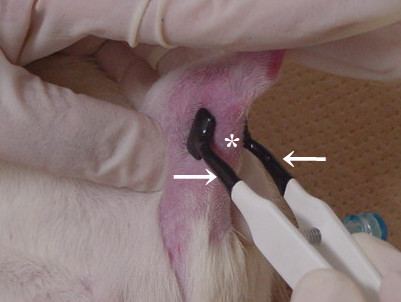
**Transcutaneous *in vivo *electroporation for skeletal muscle**. After the injection of 25 μg of plasmid into the gastrocnemius muscle, transcutaneous *in vivo *electroporation was performed via the skin of the calf to drive the gene transfer. The conditions were 8 pulses of 100 V, 50 msec. *, plasmid-injected area; arrows, electrodes.

Rats (N = 60) were anesthetized by intraperitoneal injection of pentobarbital sodium (5.0 mg/100 g of body weight). The fur on the target area of the leg was removed with clippers. As shown in Fig 1, plate electrodes (Nepa Gene, Chiba, Japan) consisting of pairs of stainless steel plates with a fixed length of 5 mm were attached to the skin at the target site after being coated with keratin cream (Fukuda Denshi, Tokyo, Japan). The accuracy of the electric current applied was confirmed by measuring the resistance (usually below 800 Ω) between the electrodes, which surrounded the middle of the gastrocnemius muscle. Next, 50 μl of plasmid DNA (total 25 μg): 12.5 μg pCAGGS and 12.5 μg pCAGGS-BMP-2, 12.5 μg pCAGGS and 12.5 μg pCAGGS-BMP-7, 12.5 μg pCAGGS-BMP-2 and 12.5 μg pCAGGS-BMP-7, or 25 μg pCAGGS was injected with a 30-gauge needle into the center of the muscle between the electrodes. Electroporation was started immediately after the injection by applying 8 electrical pulses (100 V, 50 msec): four square pulses followed by 4 more of the opposite polarity at 1-sec intervals using an electroporator (CUY21EDIT; Nepa Gene).

### Reverse transcription (RT)-PCR

Rats (each group N = 5) that had received injections of pCAGGS and pCAGGS-BMP-2, pCAGGS and pCAGGS-BMP-7, pCAGGS-BMP-2 and pCAGGS-BMP-7, or pCAGGS alone were killed under general anesthesia and the target muscles were resected (0.15 g) at 1, 3, 5, 7, and 9 days after gene transfer. Total RNA was isolated from the targeted muscle using Isogen (Nippon Gene, Tokyo, Japan). mRNAs for human BMP-2, human BMP-7, rat BMP-4, and glyceraldehyde-3-phosphate dehydrogenase (G3PDH) were detected by RT-PCR using the following primers: human BMP-2 backward primer, 5'-TCTCTGTTTCAGGCCGAACA-3'; human BMP-2 forward primer, 5'-TCTGACTGACCGCGTTACTC-3'; human BMP-7 backward primer, 5'-CAAGCCCAAAATGGAGAGGA-3'; human BMP-7 forward primer, 5'-TCTGACTGACCGCGTTACTC-3'; rat BMP-4 backward primer, 5'-TTCTCCAGATGTTCTTCGTG-3'; rat BMP-4 forward primer, 5'-ACTGCCGCAGCTTCTCTGAG-3'; G3PDH backward primer, 5'-TCCACCACCCTGTTGCTGTA-3'; G3PDH forward primer, 5'-ACTGGCGTCTTCACCACCAT-3'. The human BMP-2 and BMP-7 forward primers were designed to hybridize with the sequence immediately downstream of the transcriptional start site of the CAG promoter, so the PCR products were not contaminated by plasmid or genomic DNA. RNA (1 μg) was incubated at 42°C for 1 hr in a total volume of 20 μl and then at 94°C for 3 min, followed by 40 cycles of 94°C for 1 min, 55°C for 1 min, and 72°C for 1 min. The PCR products were analyzed by 2% agarose gel electrophoresis to detect the 285-bp human BMP-2 mRNA, 279-bp human BMP-7 mRNA, 546-bp rat BMP-4 mRNA, and 682-bp G3PDH mRNA.

### Radiographic analysis

Ten days after the gene transfer, the rats (each group N = 6) were sacrificed with an overdose of sodium pentobarbital. The injected regions of the calf muscles were excised and analyzed with a soft X-ray to detect calcified areas (SRO-M50, Saffron Inc., Tokyo, Japan).

### Histological and immunohistochemical analyses

Specimens (each group N = 1) obtained 1, 3, 5, 7 and 10 days after electroporation were fixed with 0.05 M phosphate-buffered 4% paraformaldehyde (pH 7.4), embedded in paraffin without decalcification, cut into 4-μm-thick sections, and then stained with hematoxylin and eosin (HE), and von Kossa stain. For immunohistochemical detection of BMP-4, serial sections were rehydrated and immersed in 0.5% periodic acid solution for 10 min to eliminate the non-specific reaction by endogenous peroxidase, then incubated in PBS containing 10% bovine serum albumin for 15 min, and washed with PBS. They were then incubated with an anti-BMP-4 monoclonal antibody (Novocastra laboratories, Newcastle, United Kingdom) diluted 1:400. Antibody incubation was carried out for 12 h at 4°C, and the sections were rinsed several times before incubating them with peroxidase-labelled secondary antibody (Sigma, St. Louis, USA) diluted 1:200 for 1 h at room temperature. The incubation was terminated by washing the sections with PBS. The sections were then immersed in a medium that consisted of 3, 3'– diaminobenzidine tetrahydrochloride (20 mg), 30% H_2_O_2_, (10 μl), and 0.05 M Tris-HCl buffer (pH 7.6) (100 ml) for 10 min at room temperature.

### Statistical analysis

Results are presented as the mean ± standard error of the mean (SEM). The statistical analysis of differences in the ALP activity among the groups was performed by analysis of variance (ANOVA), followed by Fisher's comparison test.

## Results

### ALP activity in C2C12 cells

**Figure 2:**
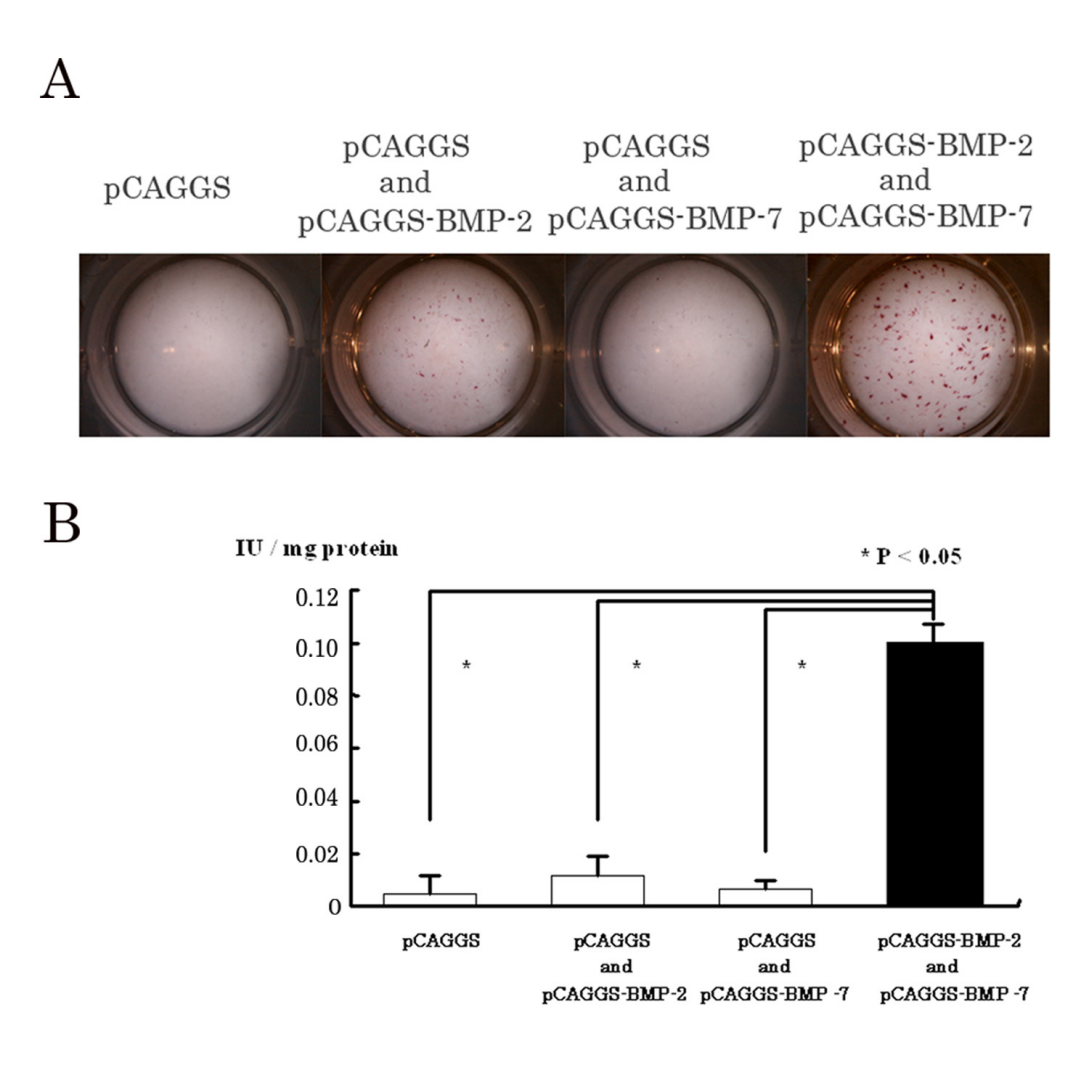
**Simultaneous gene transfer with BMP-2 and BMP-7 *in vitro***. ALP activity in C2C12 cells transfected with pCAGGS, pCAGGS and pCAGGS-BMP-2, pCAGGS and pCAGGS-BMP-7, or pCAGGS-BMP-2 and pCAGGS-BMP-7. ALP staining in the C2C12 cells 10 days after the treatment (A). There were many more ALP-positive clusters in the combined (BMP-2 and BMP-7) gene transfer group than in cultures transfected with either gene alone. Quantitative analysis of the ALP activity in C2C12 cells transfected with BMP-2, BMP-7, or the combined BMP-2 and BMP-7 genes (B). A more than five-fold increase in ALP activity was seen after gene transfer with pCAGGS-BMP-2 and pCAGGS-BMP-7 compared with pCAGGS-BMP-7 or pCAGGS-BMP-2 alone, respectively. (*P < 0.05.)

Ten days after the gene transfer by lipofection, the C2C12 cells that had been directly and simultaneously transfected with equal doses of pCAGGS-BMP-2 and pCAGGC-BMP-7 showed stronger ALP activity by histochemistry than cells transfected with the BMP-2 gene alone (Fig. 2A). In contrast, almost no positive cells were seen in the C2C12 cells transfected with pCAGGS-BMP-7 or pCAGGS alone. Quantitative analysis of the ALP activity revealed that the activity in the pCAGGS-BMP-2 and pCAGGS-BMP-7-treated group was more than 5 times greater than in the single-gene transfer or control groups (p < 0.05) (Fig. 2B).

### Human BMP-2 and BMP-7 mRNA expression in muscles electroporated with pCAGGS-BMP-2 and pCAGGS-BMP-7

**Figure 3:**
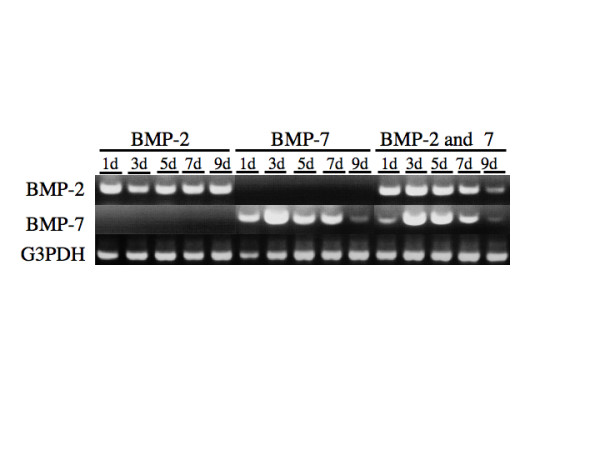
**RT-PCR evaluation of BMP-2 and BMP-7 gene expression after simultaneous gene transfer into skeletal muscles**. RT-PCR of human BMP-2 or BMP-7 mRNA in muscle after transfection by transcutaneous electroporation with pCAGGS and pCAGGS-BMP-2, pCAGGS and pCAGGS-BMP-7, or pCAGGS-BMP-2 and pCAGGS-BMP-7.

To evaluate whether the human BMP-2 and -7 genes could be simultaneously and directly transferred by *in vivo *electroporation, we analyzed the BMPs' mRNA expression in the electroporated muscles by RT-PCR. The presence of human BMP-2 or BMP-7 mRNAs transcribed from pCAGGS-BMP-2 or pCAGGS-BMP-7 in the electroporated muscles was detected 1,3,5,7 and 9 days after treatment (Fig. 3). Both human BMP-2 and BMP-7 mRNAs were co-expressed in muscles after the simultaneous transfer of pCAGGS-BMP-2 and pCAGGS-BMP-7 using transcutaneous *in vivo *electroporation up to 9 days. The G3PDH mRNA control was detected in all groups. These data revealed that multiple BMP genes could be successfully transferred by *in vivo *electroporation.

### Simultaneous and direct electroporation-mediated *in vivo *gene transfer of pCAGGS-BMP-2 and pCAGGS-BMP-7 induces more rapid ossification than the transfer of either gene alone

The induction of bone formation was investigated by radiographic and histological observations 10 days after the gene transfer.

#### Radiographic findings

**Figure 4:**
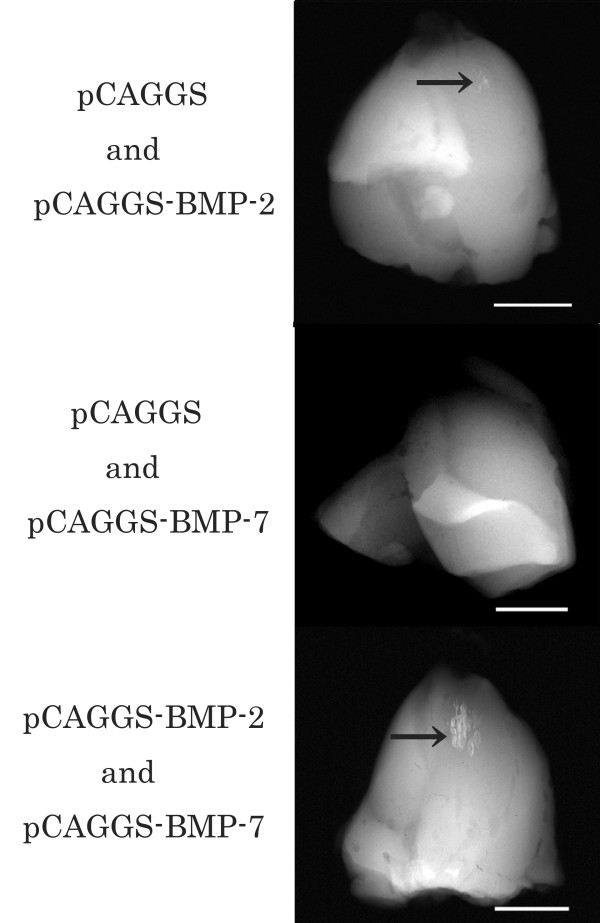
**Soft X – ray analysis 10 days after *in vivo *electroporation**. Soft X-ray images of target muscles on day 10 after gene transfer with pCAGGS and pCAGGS-BMP-2, pCAGGS and pCAGGS-BMP-7, or pCAGGS-BMP-2 and pCAGGS-BMP-7. Radio-opaque areas were seen after electroporation with pCAGGS and pCAGGS-BMP-2 (arrow) or pCAGGS-BMP-2 and pCAGGS-BMP-7 (arrow). No opacities with well-defined margins were detected in muscles that received pCAGGS and pCAGGS-BMP-7 by electroporation. Scale bar: 10 mm.

Radiographs revealed opacities that had well-defined margins in the target muscles of groups receiving electroporation with pCAGGS and pCAGGS-BMP-2, and pCAGGS-BMP-2 and pCAGGS-BMP-7. The opaque areas in the muscles treated simultaneously with the BMP-2 and BMP-7 genes were larger and clearer than those in the other group (Fig. 4). In contrast, we could not detect well-defined opacities in muscles that received transferred pCAGGS-BMP-7 and pCAGGs or pCAGGS alone.

#### Histological findings

**Figure 5:**
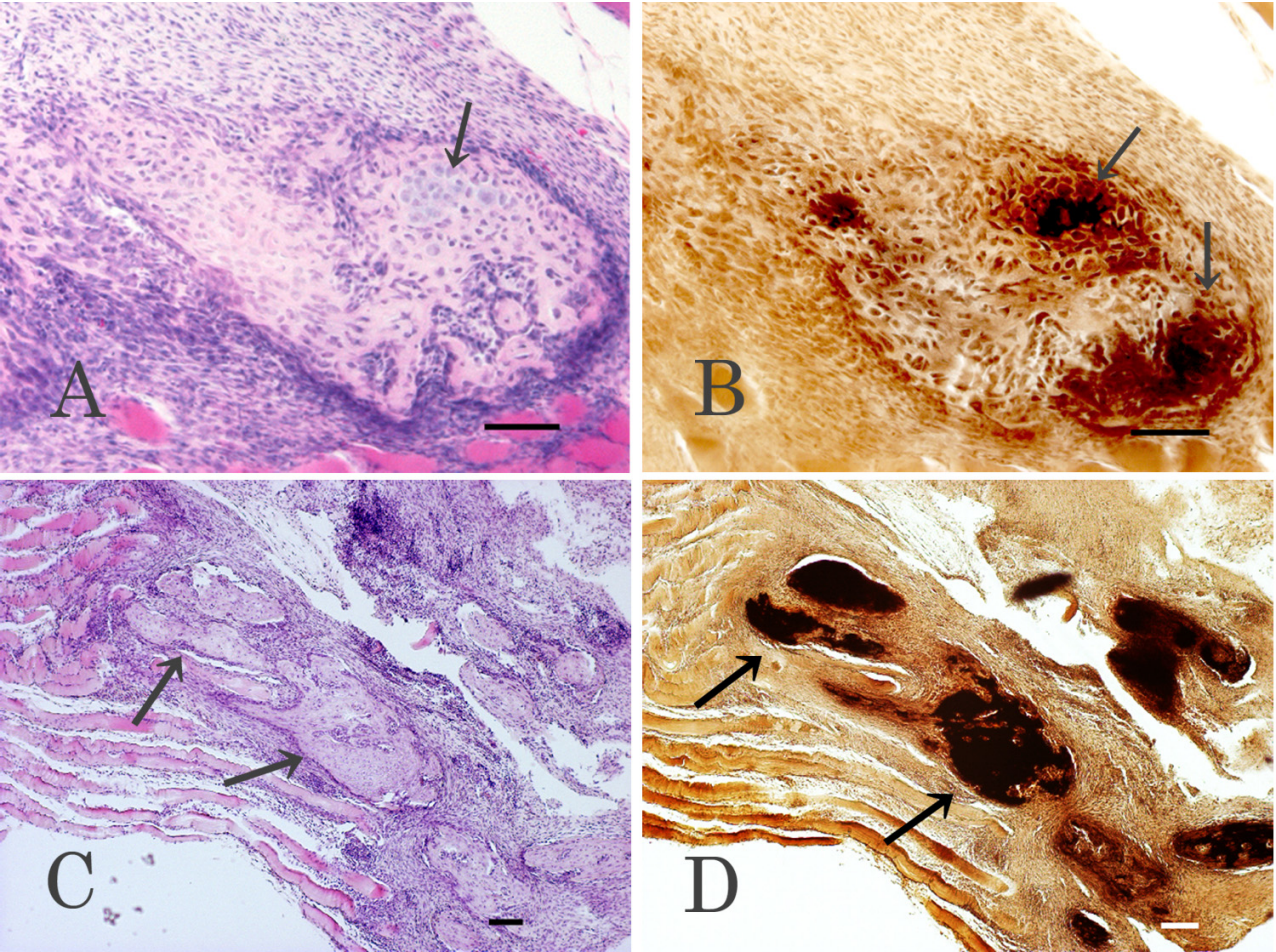
**Histological analyses 10 days after *in vivo *electroporation**. Histological examinations were carried out 10 days after electroporation with pCAGGS and pCAGGS-BMP-2 or pCAGGS-BMP-2 and pCAGGS-BMP-7. A section stained with HE from muscle electroporated with pCAGGS and pCAGGS-BMP-2 (A) contained cartilage (arrow). A serial section stained with von Kossa showing partially calcified areas (B, arrows). A HE-stained section of muscle electroporated with pCAGGS-BMP-2 and pCAGGS-BMP-7 showing ectopic bone (C, arrows). The calcified areas that stained with von Kossa (D, arrows) in the pCAGGS-BMP-2 and pCAGGS-BMP-7-transfected muscles were much wider and more strongly stained than in the BMP-2-transfected sections (B). Scale bar: 100 μm.

We examined sections from muscles harvested 1, 3, 5, 7, or 10 days after the electroporation histologically, by HE and von Kossa staining. By 10 days after the treatment, both cartilaginous tissues and bone had formed in specimens treated with pCAGGS and pCAGGS-BMP-2 or pCAGGS-BMP-2 and pCAGGS-BMP-7. However, in specimens from rats that received pCAGGS-BMP-2, the cartilaginous regions, rather than ossified regions, were predominant and widely distributed. Partially calcified areas were also observed by von Kossa stain (Fig. 5A,B). In contrast, bone was frequently observed among muscle fibers in the pCAGGS-BMP-2 and pCAGGS-BMP-7-treated specimens (Fig. 5C), and the bone contained osteoblasts, osteocytes, and osteoclasts. In addition, calcification was further advanced in these pCAGGS-BMP-2 and pCAGGS-BMP-7-treated specimens than in the pCAGGS-BMP-2-treated specimens, as revealed by von Kossa stain (Fig. 5D). Neither cartilaginous tissue nor bone was observed in the specimens treated with pCAGGS or pCAGGS and pCAGGS-BMP-7, 10 days after treatment.

### Expression of rat endogenous BMP-4 mRNA

**Figure 6:**
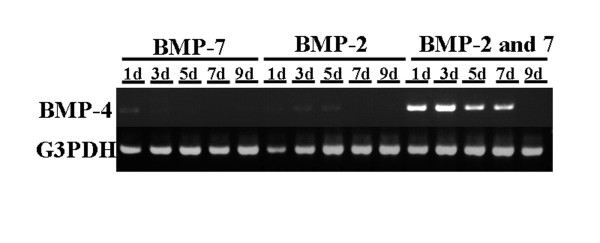
**Rat BMP-4 expression after gene transfer with BMP-2, BMP-7, or BMP-2 and BMP-7**. Rat BMP-4 mRNA expression in muscles electroporated with pCAGGS-BMP-2, pCAGGS-BMP-7, or pCAGGS-BMP-2 and pCAGGS-BMP-7 detected by RT-PCR. The BMP-4 mRNA was highly expressed in the muscles that received the combined BMP-2 and BMP-7 gene transfer.

In the group treated with the combined BMP-2 and BMP-7 genes, rat BMP-4 mRNA was expressed, and its level increased up to 3 days after gene transfer in a time-dependent manner. Its expression gradually decreased thereafter, and disappeared by 9 days after the gene transfer (Fig. 6). Rat BMP-4 mRNA expression was weak in the BMP-2 and in the BMP-7 single-gene transfer groups.

### Immunohistochemical reactivity of BMP-4

**Figure 7:**
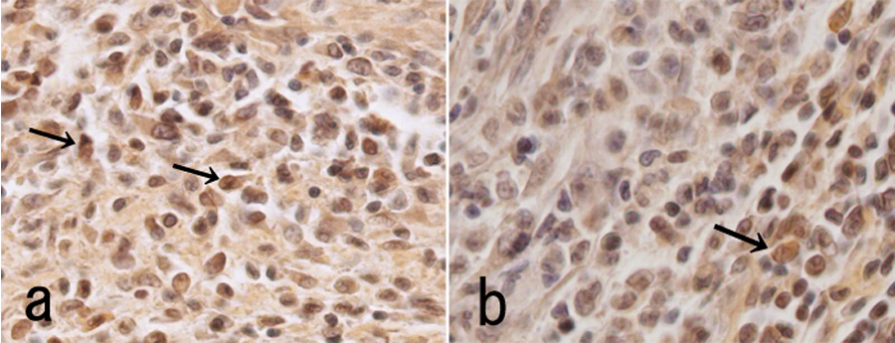
**Immunohistochemical analysis of BMP-4 in skeletal muscle**. Immunohistochemical detection of BMP-4-positive cells 5 days after the combined transfer of the BMP-2 and BMP-7 genes (a), or the BMP-2 gene alone (b). The positive cells (arrows) were numerous in the matrix between the muscle fibres in the muscle transfected with the combined BMP genes compared with muscle transfected with BMP-2 alone.

The distribution of BMP-4-positive cells showed that, compared with specimens treated with single-gene transfer, there were higher numbers of positive cells in specimens treated with both the BMP-2 and BMP-7 genes. The BMP-4-positive cells were located in the matrix between the muscle fibers, which was greatly expanded in these specimens (Fig 7).

## Discussion

In the present study, we evaluated the effect of the direct and simultaneous gene transfer of human BMP-2 and BMP-7 gene expression vectors (pCAGGS-BMP-2 and pCAGGS-BMP-7) on osteoinduction using *in vivo *electroporation.

*In vitro *studies showed previously that BMP purified from the conditioned medium of cultured CHO cells that were transfected with equal amounts of BMP-2 and BMP-7 expression vectors increases the ALP activity of mouse stromal cells far better than BMP purified from the medium of cells transfected with one of these vectors alone. These results suggest that a BMP-2/7 heterodimer forms under these conditions and causes the high ALP activity, indicating that the BMP-2/7 heterodimer has a strong bone-inducing activity [9,10,16]. Furthermore, mixing the conditioned media from cells secreting either BMP-2 or BMP-7 alone does not increase the ALP activity synergistically [10,16]. These systems had three steps: the first was the combined gene transfer of BMP-2 and BMP-7 into epithelial cells to produce BMP-2/7. Next the BMP2/7 protein was purified from the supernatant of these cells. Finally, the purified protein was added to the preosteoblastic or myoblastic cells. Here, we assessed the effect of the direct and simultaneous gene transfer of BMP-2 and BMP-7 into skeletal muscles. Before our *in vivo *study, we carried out an *in vitro *experiment using the mouse myoblastic C2C12 cell line to determine whether cells directly and simultaneously transfected with the BMP-2 and BMP-7 genes would express these genes, as we hoped they would *in vivo*. In the present study, the C2C12 cells showed higher ALP activity in the group receiving the BMP-2 and BMP-7 genes than in the control group or the groups transfected with a single BMP expression plasmid. These results agreed with the previous studies [9,10].

Based on our *in vitro *study, we examined whether *in vivo *electroporation with equal doses of pCAGGS-BMP-2 and pCAGGS-BMP-7 is effective for bone formation. Ten days after treatment, direct and simultaneous transfer of the BMP-2 and BMP-7 genes induced higher ALP activity (data not shown), and more intense calcification than did the transfer of either gene alone. In contrast, the calcified areas observed 10 days after the gene transfer with pCAGGS-BMP-2 alone were mostly cartilaginous tissue with very little bone. The muscles transfected with pCAGGS-BMP-7 did not form cartilaginous tissue, bone, or calcified areas during the observation period. Therefore, this study strongly suggests that the direct and simultaneous gene transfer of equal doses of pCAGGS-BMP-2 and pCAGGS-BMP-7 into skeletal muscles by *in vivo *electroporation was successful and resulted in greater osteoinductive activity than the direct transfer of either gene alone. This effect may be owing to the formation of the BMP-2/7 heterodimer as suggested for the *in vitro *studies [9,10,16]. However, to fully understand our results, we may need to consider the more complicated and dynamic events underlying osteoinduction *in vivo*, because the extracellular matrix components that bind to BMPs [17] and/or the different affinities of the BMPs for their receptors [18-21] must be involved in the osteoinductive activity. Further study is needed to clarify the mechanism of the osteoinduction reported here. For example, a single vector that can express BMP-2 and BMP-7 simultaneously might be useful for assessing the influence of the BMP-2/7 heterodimer on osteoinductive activity *in vivo*.

Endogenous BMP-4 mRNA was highly expressed in the skeletal muscle of rats receiving the pCAGGS-BMP-2 and pCAGGS-BMP-7 genes, but its expression was much weaker in the skeletal muscles of rats receiving only one of the genes. It is reported that BMP-7 down-regulates BMP-4 mRNA through the stages of proliferation, matrix formation, and mineralization in long-term primary cultures of fetal rat calvarial (FRC) cells [14]. In C2C12 cells, BMP-7 up-regulates the BMP-4 mRNA [22]. On the other hand, BMP-2 inhibits BMP-4 mRNA expression initially, but stimulates it during the mineralization phase [11,13]. In the costochondral growth-plate chondrocytes, BMP-2 induces the expression of BMP-4 mRNA in a cell maturation-dependent manner [12]. However, this is the first report that the simultaneous gene transfer by *in vivo *electroporation of BMP-2 and BMP-7 increases the level of BMP-4 mRNA.

Under conditions of normal osteoblastic cell differentiation, rat BMP-4 mRNA is expressed in the early phase with peak expression levels during the proliferation and matrix formation phases, but it declines afterwards in FRC cells [10,23]. *In vivo*, a transient elevation in the BMP-4 mRNA levels is reported in osteoprogenitor cells of the periosteum in the early stages of fracture repair [24-27]. Moreover, BMP-4 mRNA is detected in areas where fibroblast-like cells and preosteoblasts migrate, in the early stage of distraction osteogenesis [28]. These results seem to suggest that BMP-4 plays an important role in the early phase of osteoinduction. Our histological results at 3 and 5 days, during the early phase of the BMP-4 mRNA increase, revealed abundant migratory spindle-shaped cells, i.e., fibroblast-like cells. These cells were in the same location as those that stained positive for BMP-4 by immunohistochemistry. Therefore, the expression of BMP-4 mRNA and its increase in the early phase might be involved with the effective bone formation seen when the BMP-2 and BMP-7 genes were simultaneously transferred into rat skeletal muscles by transcutaneous *in vivo *electroporation.

In this study, we used an animal model to study ectopic bone formation in the skeletal muscles to examine the efficiency of simultaneous and direct BMP-2 and BMP-7 gene transfer using *in vivo *electroporation. For clinical applications, it may be possible to reconstruct bone and the accompanying muscle in jaw areas that have been damaged, for example, by trauma or tumour treatment. Moreover, BMP-2 and BMP-7 gene transfer into the periostea to augment the alveolar ridge or fracture repair may enhance periosteal cell differentiation efficiently, because it is well known that periostea have multi-potential stem cells [29-31]. If successful, such clinical applications would be very significant and wide-ranging, and could include fracture repair, orthognathic surgery, alveolar ridge augmentation, and dental implants.

## Conclusion

We propose that the direct and simultaneous gene transfer of BMP-2 and BMP-7 by *in vivo *electroporation is more effective than the transfer of BMP-2 alone, which we explored in an earlier paper using the same conditions, such as the dose of plasmid and the size of the electrodes [5]. Since the major drawbacks of using adenovirus vectors, i.e., the need for titration, the potential toxicity, and the need to suppress the host immune response, are not necessary using this system, gene therapy via *in vivo *electroporation is safer and simpler than via viral vectors. As a methodological consideration, electroporation is quite suitable for the direct and simultaneous transfer of two or more BMP genes for bone regeneration therapy.

## Competing interests

The author(s) declare that they have no competing interests.

## Authors' contributions

MK performed all experiments, analyzed the data, and prepared the manuscript. KB and TY contributed to the *in vivo *electroporation experiments and provided useful discussions about the results. HM and JM provided significant help to MK in constructing the plasmid vector.

## Pre-publication history

The pre-publication history for this paper can be accessed here:
